# The Story of New York Briefly Told

**Published:** 1899-11

**Authors:** 


### THE STORY OF NEW YORK BRIEFLY TOLD.

(Continued from page 669, October number.)

THE Wednesday morning session was called to order rather late, owing to the fact that many of the members were present at the clinics of the American Horse Exchange, which had been kindly placed at the use of the Association by the Board of Directors, and the kindness of Mr. Ware and his assistants added much to the value and thoroughness with which these were conducted, the place affording all that could be desired, so far as cleanliness, light, and convenience, points which were very much appreciated by the operators and those witnessing the clinical demonstrations.

Dr. W. L. Williams performed the operation of vaginal ovariotomy in a thoroughbred filly, briefly explaining the *modus operandi.* The time consumed in performing the same was six minutes. The animal seemed, the next morning, little the worse of the operation. The veterinary operating-table which was used for several of the operations was explained and its workings demonstrated, and the salient points of value were elucidated by Dr. Williams.

Median neurectomy was performed by Dr. C. E. Clayton. The removal of the lateral cartilage for the cure of quittor was performed by Dr. George H. Berns. This operation was done under anæsthesia, and the patient was removed from the table while still under the influence of the anæsthetic.

Wednesday morning being the time set for the election of officers, a happy situation seemed to prevail. The candidates presented were without opposition, and they seemed to be the unanimous choice of the various members, resulting in the election for President, Dr. Leonard Pearson, of Philadelphia; Dr. J. F. Winchester, of Lawrence, Mass., Eastern Vice-President; Dr. S. Brenton, of Detroit, Michigan, Central Vice-President; Dr. H. L. Ramacciotti, of Omaha, Nebraska, Western Vice-President. The re-election of Dr. S. Stewart as Secretary followed with the hearty concurrence of all, likewise the selection of Dr. Wm. Herbert Lowe as Treasurer.

Again it was found necessary to limit papers to twenty minutes, many of which were cut off in the most interesting part, that of "Dietetics," by Dr. Dalrymple, and "Disinfection," by Dr. E. A. A. Grange, were very quickly disposed off, to the loss of every member present when two such important topics were under consideration.

"Routine Manipulations and Operations," by Dr. Wm. Herbert Lowe, was a good memory-jogger, and made many recall how quickly some things are forgotten that should not be.

"A Plea for the More General Use of Anæsthesia," by Dr. J. P. Turner, was a timely one, for the discussion of this paper awakened some marked differences of opinion, especially in regard to several of the everyday operations on the equine and canine species. The subsequent sickness from the use of anæsthetics for many hours after operation was commented on by several, and held up as a serious objection to its use, on the ground that animals seem in a great measure forgetful of pain after it has ceased to be inflicted, and resume their normal functions and habits very differently from members of the human family.

(To be continued in the December number.)

